# August Number Wanted

**Published:** 1884-01

**Authors:** 


AUGUST NUMBER WANTED.

In consequence of the unexpected call for back numbers of this journal, the edition for August, 1883, has been exhausted, and we have not sufficient for our files. Many of our readers must have copies which they do not care to preserve. If they will send them to the editor they will confer a great favor upon him, and a reasonable sum will be paid for them, or copies of other numbers will be sent in exchange.

